# Evenamide: A Potential Pharmacotherapeutic Alternative for Treatment-Resistant Schizophrenia

**DOI:** 10.1093/ijnp/pyae005

**Published:** 2024-01-09

**Authors:** Raghunath Singh, Margaret K Hahn, Yashika Bansal, Sri Mahavir Agarwal, Gary Remington

**Affiliations:** Schizophrenia Division, the Centre for Addiction and Mental Health, Toronto, ON, Canada; Schizophrenia Division, the Centre for Addiction and Mental Health, Toronto, ON, Canada; Institute of Medical Sciences, University of Toronto, Toronto, ON, Canada; Department of Psychiatry, University of Toronto, Toronto, ON, Canada; Banting and Best Diabetes Centre, University of Toronto, Toronto, ON, Canada; Neurobiology of Depression and Aging Lab, the Centre for Addiction and Mental Health, Toronto, ON, Canada; Schizophrenia Division, the Centre for Addiction and Mental Health, Toronto, ON, Canada; Institute of Medical Sciences, University of Toronto, Toronto, ON, Canada; Department of Psychiatry, University of Toronto, Toronto, ON, Canada; Banting and Best Diabetes Centre, University of Toronto, Toronto, ON, Canada; Schizophrenia Division, the Centre for Addiction and Mental Health, Toronto, ON, Canada; Institute of Medical Sciences, University of Toronto, Toronto, ON, Canada; Department of Psychiatry, University of Toronto, Toronto, ON, Canada

Schizophrenia (SCZ) is a complex psychiatric syndrome affecting over 1% of the world population and a leading cause of global disability (Marder and Cannon, 2019). Antipsychotics (APs) are the cornerstone of treatment in SCZ and generally effective in treating symptoms of psychosis. However, a significant proportion (nearly one-fifth to one-half) of individuals with SCZ remain unresponsive to AP treatment. Treatment-resistant schizophrenia (TRS) is defined as a persistent occurrence of positive symptoms following 2 trials of adequate (dose and duration) AP treatment (Howes et al., 2017; Potkin et al., 2020). Onset of TRS may occur during the first episode of psychosis or may develop later in the illness (Potkin et al., 2020). TRS patients have an increased risk of mortality and morbidity and usually require frequent hospitalization, adding to societal economic burden and cost (Kadakia et al., 2022; Anand et al., 2023). Clozapine remains the only agent approved to treat TRS, although it remains markedly underutilized secondary to its side effects profile, in particular agranulocytosis and the associated requirement of routine bloodwork in most countries (Marder and Cannon, 2019). Alongside other SGAs, clozapine is also associated with inducing severe metabolic side effects, such as weight gain and type 2 diabetes, cardiovascular complications (QTc prolongation), gastroparesis, and risk of seizure (Yuen et al., 2018). Furthermore, nearly 60% of the TRS patients demonstrate a suboptimal response to clozapine (Siskind et al., 2017). Electroconvulsive therapy (ECT) has been beneficial as an augmentation strategy in clozapine-resistant SCZ, while evidence related to the addition of other antipsychotic agents remains equivocal (Potkin et al., 2020).

Lack of options to currently manage TRS and clozapine’s limited efficacy have encouraged ongoing work addressing treatment options. One such line of investigation has focused on glutamatergic or N-methyl-d-aspartate receptor (NMDAR) hypofunction in SCZ. Nonresponders with first episode of psychosis, as well as individuals with TRS, have been shown to demonstrate normal dopamine synthesis (Kim et al., 2017) but higher glutamate levels in the anterior cingulate cortex (Jauhar et al., 2018), encouraging the pursuit of agents targeting NMDAR.

Evenamide, formerly NW-3509 and developed by Newron Pharmaceuticals, is a chemical moiety that inhibits voltage-gated sodium channels and normalizes the excessive synaptic glutamate (without affecting basal levels) produced as a result of NMDAR hypofunction. In doing so, evenamide reduces cortical and hippocampal hyperexcitability without affecting other neurotransmitter systems, which may be implicated in various adverse side effects (Anand et al., 2017; Singh et al., 2019). Its efficacy has been examined in several preclinical models, such as ketamine-induced disrupted pre-pulse inhibition (Bortolato et al., 2018), as well as phencyclidine-induced social deficits (Faravelli et al., 2016). Evenamide alone or in combination with clozapine significantly restored ketamine-induced pre-pulse inhibition deficits in rats (Bortolato et al., 2018). Clinical studies have also supported both efficacy and safety of evenamide (Singh et al., 2019). For example, in a preliminary double-blind, placebo-controlled randomized controlled trial involving 89 patients with mild to moderately severe SCZ, 4 weeks of evenamide (15–25 mg b.i.d.) in combination with risperidone or aripiprazole significantly improved Positive and Negative Syndrome Scale (PANSS) total and positive subscale scores, as well as Clinical Global Impression (CGI) scale scores. It was well tolerated, although adverse events in 2 patients included atrial fibrillation and seizure, respectively, which require further investigation (Anand et al., 2018).

Based on these pilot data (Anand et al., 2018), this recently published phase-II clinical trial evaluated long-term efficacy and safety of evenamide as an add-on to existing AP (except clozapine) treatment in patients meeting criteria for TRS (Anand et al., 2023). Treatment response and resistance in psychosis guidelines were used for the diagnosis of TRS (Howes et al., 2017). At baseline, patients had moderate to severe illness (CGI-S between 4 and 6), predominant positive symptoms (score of ≥4), and functional deficits. In 161 patients, the mean duration of illness was 6.8 years, while olanzapine and risperidone were the most commonly prescribed APs (81.3%), with the mean daily dose of 21.7 mg and 7.5 mg, respectively. In all patients (n = 156) at 6 weeks, the PANSS total score was significantly reduced (11.6% from baseline to sixth week). In patients who completed 1 year of the study (n = 97), improvement in the PANSS total score remained significant at 6 months (15.9%) and 1 year (18.6%). Similarly, the proportion of PANSS “responders” (≥20% improvement in total PANSS score) also increased from 16.5% (at week 6) to 39.2% (at month 6) to 47.4% (at 1 year). In “responders,” clinically significant efficacy of evenamide was also observed in terms of CGI severity of illness (CGI-S 16.5% at 6 months, 28.9% at 1 year) and CGI change from the baseline (CGI-C 36.1% at 6 months, 41.2% at 1 year). Again, over the 1-year treatment duration, evenamide was well tolerated. Two patients discontinued due to adverse events (influenza-like symptoms and somnolence). Other treatment-emergent adverse events included dizziness, insomnia, and pyrexia (in 4 patients) (Anand et al., 2023).

Despite these early promising findings, the current study has several limitations: (1) it is not a placebo-controlled or double-blind study, and the majority of the patients enrolled were from 1 country (India), which limits the finding’s generalizability; (2) positive symptoms are often defined as the core features of TRS, although the current study did not report on this domain specifically, instead presenting data on total PANSS score changes only; and (3) other findings cited (Anand et al., 2017, 2018) represent conference abstracts, which are not peer reviewed or do not provide access to the full data set. In addition, the results from the open label study showing overall improvement in symptoms and safety of evenamide are superficially impressive but comparable to Lu AF35700, which failed to separate from an active comparator (risperidone/olanzapine) (Kane et al., 2022). This further highlights the importance of evidence established through a double-blind design.

Taken together, the long-term efficacy and safety of evenamide warrant a global (multicentric), double-blind, placebo-controlled, randomized clinical trial. Replication of the current results, including comparison with the sole approved treatment for TRS (clozapine), would favorably position this line of investigation as a novel approach in the psychopharmacotherapy of TRS.

## Data Availability

No new data was generated or analyzed to support this research.

## Interest Statement

G.R. has received a consultant fee from HLS Therapeutics Inc. M.K.H. has received Alkermes consultant fees. S.M.A. has served as consultant for HLS Therapeutics and on the Ad-board for Boehringer Ingelheim, Canada.
